# Quantification of myocardial oxygen consumption with ^17^O-CMR: initial study

**DOI:** 10.1186/1532-429X-13-S1-P345

**Published:** 2011-02-02

**Authors:** Jie Zheng, David Muccigrosso, Dana Abendschein

**Affiliations:** 1 Washington University In St. Louis, Saint Louis, MO, USA

## Background

Impaired myocardial oxygenation leading to ischemia is central to the pathophysiology of coronary artery disease and an important contributor to other common cardiovascular disease conditions such as left ventricular hypertrophy, dilated cardiomyopathy, renal heart disease and valvular heart disease. Positron Emission Tomography (PET) can quantify myocardial oxygen consumption (MVO_2_), but has limited applications due to its relatively low spatial resolution, high cost, and ionizing radiation.

## Purpose

This study is aiming to develop a new non-invasive CMR method with the use of ^17^O based blood tracer to assess myocardial oxygenation and MVO_2_.

## Methods

The initial ^17^O-CMR was performed in normal mongrel dogs (n=3) and myocardial ischemic dogs (n=3). The later dogs includes 70% (n=1) and 100% (n=2) area stenosis in the second diagonal branch of the left descending coronary artery (LAD). The ^17^O blood tracer was prepared using artificial blood perfluorodecalin emulsion or PFD (OxyToT, Rockland Technimed Ltd, Airmont, NY) and 70% enriched ^17^O_2_ gas. Each dog was injected 2 mL/kg ^17^O-PFD at rest within 30 sec. A novel CMR T_1ρ_-weighted imaging was performed at baseline and then to monitor the myocardial T_1ρ_ signals for over 30 min after the injection. The ^17^O_2_ gas absorbed in PFD will be taken up by the myocardial tissue and converted into H_2_^17^O water, which will be detected by T_1ρ_-weighted imaging with a negative contrast. The H_2_^17^O water concentration can be obtained with the ratio between T_1ρ_-weighted signals after and before the ^17^O-PFD injection. MVO_2_ can be quantified using a new model developed in our laboratory. Quantitative perfusion CMR imaging was also performed at the end of study to confirm the ischemic area.

## Results

**Figure 1:**
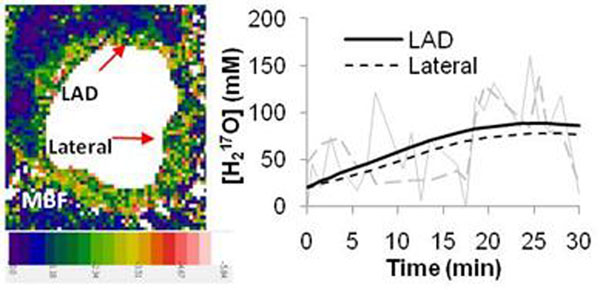
Quantitative myocardial perfusion map of a 70% stenosis (left panel). The converted [H_2_^17^O] curve (right panel) shows elevated [H_2_^17^O] levels in LAD and suppressed [H_2_^17^O] in lateral region. The lines in this plot indicate fitted data from our model.

**Figure 2:**
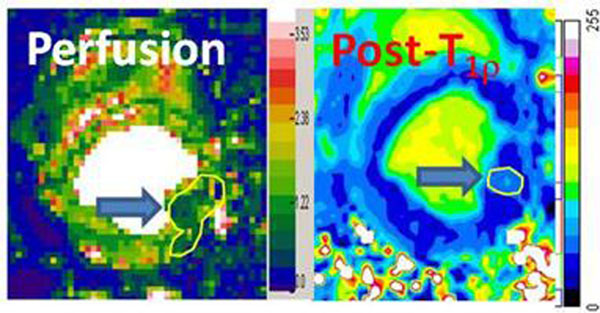
A 100% stenosis resulted in perfusion deficit in lateral wall (arrow on perfusion map). Resting T_1p_-weighted ratio image shows less signal drop in lateral wall after injection of ^17^O-PFD (arrow in post-T_1p_) compared to signals in the rest of the myocardium. The deficit area in the post-T_1p_ image (yellow circle) is much smaller than the hypo-perfusion area in the perfusion map (yellow ROI).

The averaged MVO_2_ in the anterior myocardial region of three normal dogs was 3.96 ± 0.97 μmol/g/min, which agrees well with MVO_2_ measured by PET in mongrel dogs. For the 70% stenotic dog, the MVO_2_ was 2.84 μmol/g/min in the anterior region (normal LAD perfused segment) and 1.57 μmol/g/min in the lateral region (the diagonal branch of LAD perfused segment), respectively (Figure 1). Figure 2 shows quantitative perfusion map and a post-T1p-weighted image in a dog with the 100% stenosis, indicating that oxygen deficit area appeared to be smaller than hypo-perfusion size.

## Conclusion

The ^17^O-CMR may have potential to provide a direct and non-invasive measurement of the oxygen consumption to facilitate comprehensive evaluations of patients at molecular level with a variety of pathophysiological etiologies.

